# Secretion of DNases by Marine Bacteria: A Culture Based and Bioinformatics Approach

**DOI:** 10.3389/fmicb.2019.00969

**Published:** 2019-05-07

**Authors:** Aisha S. M. Al-Wahaibi, Emilia Lapinska, Nithyalakshmy Rajarajan, Sergey Dobretsov, Robert Upstill-Goddard, J. Grant Burgess

**Affiliations:** ^1^School of Natural and Environmental Sciences, Newcastle University, Newcastle upon Tyne, United Kingdom; ^2^Centre of Excellence in Marine Biotechnology, Sultan Qaboos University, Al Khoud, Oman; ^3^Department of Marine Science and Fisheries, Sultan Qaboos University, Al Khoud, Oman

**Keywords:** marine gel particle aggregates, bacterial diversity in the ocean, biofilm, extracellularDNA (eDNA), extracellular bacterial nuclease, protobiofilm, extracellular DNA (eDNA), marine enzymes

## Abstract

The vast majority of bacteria present in the natural environment are present in the form of aggregates and/or biofilms. Microbial aggregates are ubiquitous in the marine environment and are inhabited by diverse microbial communities which often express intense extracellular enzymatic activities. However, the secretion of an important group of enzymes, DNases, by bacteria from marine aggregates has not been studied, despite the importance of these aggregates in biogeochemical cycling of nutrients in the oceans. In this work, we therefore, employed both culture-based and bioinformatics approaches to understand the diversity of bacterial DNases in marine bacterioplankton. We found that 34% of 345 strains of attached and non-attached marine bacteria showed extracellular DNase activity. Most of these isolates belong to Proteobacteria (53%) and Firmicutes (34%). Secretion of DNases by bacteria isolated from marine gel particles (MGP) is reported here for the first time. Then, to further understand the wider diversity of the potential to produce DNases, sequences were compared using 2316 whole genome and 42 metagenome datasets. Thirty-nine different taxonomic groups corresponding to 10 bacterial phyla were found to encode genes responsible for DNase secretion. This study highlights the unexpected and widespread presence of DNase secretion in bacteria in general and in MGP more specifically. This has important implications for understanding the dynamics and fate of marine microbial aggregates in the oceans.

## Introduction

The marine environment contains an enormous microbial diversity (Salazar and Sunagawa, 2017) and density, where on average, bacteria are present in seawater and can reach concentrations of 10^6^ cells/ml (Azam, 1998; Azam and Malfatti, 2007). Bacteria can live in one of three modes: either as free-living (planktonic), attached to surfaces (biofilm), or in the form of particle aggregates (flocs). In aggregated forms, bacteria live embedded in a hydrated matrix made up of extracellular polymeric substances (EPS) in the form of biofilm or protobiofilm (Verdugo, 2007; Verdugo and Santschi, 2010; Bar-Zeev et al., 2012; Elias and Banin, 2012). Marine gel particles (MGP) is another name for suspended bacterial aggregates found ubiquitously in the oceans and which can also initiate biofilm formation when they become attached to surfaces (Bar-Zeev et al., 2012; Neukermans et al., 2016; Busch et al., 2017).

Marine bacteria can contribute to biogeochemical nutrient cycling processes by secreting extracellular enzymes (ECE) (Azam, 1998; Azam and Malfatti, 2007; Arnosti, 2011; Luo et al., 2017; Ivančić et al., 2018) which play a fundamental role in the breakdown of organic matter by the hydrolysis of high molecular weight material to low molecular weight compounds for bacterial uptake. These enzymes can also affect the integrity and stability of MGP aggregates (Vetter and Deming, 1999; Hoppe et al., 2002; Lechtenfeld et al., 2015; Balmonte et al., 2016; Liu and Liu, 2018). As a result, MGP and their associated bacterial ECE have recently attracted attention due to their important role in the process of sequestration and removal of carbon into the deep ocean during sedimentation of particles (Karner and Herndl, 1992; Grossart et al., 2007; Arnosti et al., 2012; Kellogg and Deming, 2014).

Many hydrolytic enzymes are produced by free-living bacteria and bacteria attached to marine aggregates, for instance, chitinases and proteases (Smith et al., 1992; Baltar et al., 2017). However, information on the presence and diversity of deoxyribonucleases (DNases) in marine bacteria, especially those associated with aggregates, is absent.

DNases catalyze the hydrolysis of deoxyribonucleic acid (DNA) by breaking down phosphodiester bonds (Nishino and Morikawa, 2002). Consequently, DNase is considered to have a pivotal role in DNA utilization and nutrient cycling in the environment (Mulcahy et al., 2010). In addition, extracellular DNA (eDNA) plays an important role in the formation and structure of biofilms (Whitchurch et al., 2002). Indeed, it is now considered a key structural component of the biofilm matrix (Tetz et al., 2009). eDNA plays a critical role in the attachment and stability of the biofilm matrix and DNases are now well recognized as agents which can effectively break up biofilms (Nijland et al., 2010; Jakubovics et al., 2013; Shields et al., 2013; Okshevsky et al., 2015). We were therefore interested in whether eDNA was also an important component of marine flocs, and if so, whether the secretion of DNases by floc-associated bacteria may subsequently affect the structural integrity (and sinking rates) of marine particles. The secretion of extracellular DNase has been reported in several species of marine bacteria such as *Bacillus licheniformis* (Nijland et al., 2010), *Vibrio* sp. (Maeda and Taga, 1976), *Myroides, Planococcus, Sporosarcina*, and *Halomonas* (Dang et al., 2009) although their precise role remains obscure.

The availability of eDNA as a source of nutrients in the oceans is well recognized (Dell’Anno and Danovaro, 2005) and might also explain the production of DNases. However, little is known about the diversity of DNases produced by marine bacteria generally and in marine aggregates in particular. In this study, we hypothesized that the production of extracellular DNases is common among marine bacteria. Therefore, we investigated the diversity of extracellular DNase production by free-living marine bacteria and by bacteria attached to aggregates.

In addition, to overcome the problem of culturability of environmental bacteria (Vartoukian et al., 2010), and to rapidly assess the presence of DNase genes in a wide variety of microbial species, we carried out analysis of putative DNase genes using a bioinformatics approach (Kennedy et al., 2008). This allows the discovery of enzymes from the dataset of sequences of microbial genomes to include “uncultivable” taxa (Elend et al., 2006). In addition, enzyme discovery using sequence-based databases is often faster than function-based methods (Kennedy et al., 2008) and can deepen our understanding of the diversity of extracellular enzymes in bacteria.

## Materials and Methods

### Sample Collection

Bacteria were isolated from sediment, seawater, MGP and algae samples. Sediments and seawater were collected in Nalgene bottles (Thermo scientific) from the North Sea, around 15 km off the NE UK coast (55^o^ 07 00 N 01^o^ 20 00 W), on 23/03/2015. Surface sediments from a water depth of 50 m were collected by sediment grab and seawater was collected at 5–10 m depth using a Niskin bottle mounted on a CTD (Conductivity, Temperature, Depth) frame. During R.R.S *James Cook* JC037, additional sediment samples were collected with a megacore from three stations on the Mid-Atlantic Ridge north and south-east of the Charlie-Gibbs Fracture Zone (CGFZ), 48°–54°N (water depths 2400–2750 m), between 13/07/2007 and 18/08/2007. Only cores with intact surface sediments were selected for use in this work. One core was selected from each station and sectioned at 0–5 mm (surface) and 5–10 mm (subsurface) depth horizons. The sediment subsamples were stored in 50 ml sterile Falcon tubes at -20°C prior to bacterial isolation. North Sea coastal water microbial strains were obtained from the Dove Marine Laboratory (Newcastle University). MGP were collected on 31/10/2016, in 5 m water depth about 2 km off the coast of Northumberland (55° 06 972 N 1°25 600 W). Brown *Fucus vesiculosus* and red *Palmaria palmata* algae were collected by hand at low tide on Cullercoat beach (55° 02′ N 1° 25′W) and Boulmer beach (55° 25′N 1° 34′W) in May 2011. Harvested samples were transferred immediately to the laboratory in sterile bags and on ice. The algal surface was washed with filtered seawater and bacterial samples were removed from the surface with a sterile swab. Sections of seaweed from the holdfast, apical tips and growth nodes were cut using a sterile scalpel. Biofilm from the Sea of Oman was sampled during low tide near Muscat at Ras Alhamra 23°37′17″N 58°17′13″E on 06/07/2016. Bacterial samples were removed from rocks at the intertidal zone with sterile swabs. Immediately samples were transferred to the laboratory on ice for further analysis.

### Scanning Electron Microscopy of Seaweed

For scanning electron microscopy, the seaweed samples were first fixed with a 2% (v/v) glutaraldehyde solution and kept at 4°C overnight (Dobretsov et al., 2014). Specimens were rinsed twice in 0.2 M phosphate buffer and dehydrated through a series of ethanol washes: 25, 50, 75% (30 min each) and in 100% absolute ethanol (30 min) until completely dried. They were subsequently transferred to Electron Microscopy Research Services at Newcastle University, where they were dried in a critical point dryer (Bal-tec), mounted on aluminum stubs and sputter coated with gold. Finally, biofilms were visualized at ×2000 and ×5000 magnification using a scanning electron microscope (Cambridge Stereoscan 240).

### Isolation of Bacterial Strains and Growth Conditions

Sterile swabs (Fisher, United Kingdom) were used to sample filtered particles (MGP 0.4–100 μm), biofilms and the seaweed surfaces. Swabs were streaked onto Difco Marine agar plates (BD Difco^TM^ Dehydrated Culture Media: Marine Agar 2216, United Kingdom). Sediment samples were diluted (10^-1^ to 10^-8^) in sterile phosphate buffered saline (PBS, Sigma, United Kingdom) prior to plating onto marine agar (Difco, BD, United Kingdom). Morphologically distinct isolates were sub-cultured using aseptic technique onto fresh agar plates to obtain pure single colonies. Bacterial cultures were incubated at different temperatures to verify temperature dependent DNase production as well as DNase activity and stability. Psychrophiles were cultivated at 16°C for 5 days, mesophiles at 25 or 37°C for 2 days and thermophiles at 45 or 50°C for 2 days. Isolated bacterial strains were purified and routinely grown in Marine broth (BD Difco, United Kingdom) or LB (Luria-Bertani) (BD Difco, United Kingdom) broth with agitation at 150 rpm using an orbital shaker at room temperature.

The isolation of MGP was carried out by filtration of seawater onto polycarbonate filters (0.4–100 μm) (Alldredge et al., 1993). However, due to the limitations of this method, that generally only allows subsequent analysis of the particles while attached to the filters, rather than as they occur in seawater as suspended particles, we developed a new procedure for concentrating MGPs into small water volumes as suspended particles. Seawater was pre-filtered first through a 100 μm stainless steel sterile sieve and then by gentle vacuum filtration (Glass Vacuum Filtration Device 47/50 mm, Sartorius) through 0.4 μm polycarbonate filters (PC) (Whatman, GE Healthcare Life Sciences). To reduce clogging each PC filter was used only to filter one liter of seawater. The particles remaining on the PC filters were recovered by suspending the filters in 5 ml of 0.2 μm filtered sterile seawater and gently agitating to resuspend the particles. The particles in suspension were then used for bacterial isolation. Five microliters of the particle suspension were spread onto marine agar (BD, United Kingdom) and incubated at room temperature for 1–5 days; isolation was carried out until pure strains were obtained.

### Cultivation and Identification of Extracellular DNase Producing Bacteria

All isolated bacterial strains were examined for extracellular DNase activity using DNase test agar containing methyl green dye (BD, 263220, United Kingdom). DNase production was evaluated at optimal growth temperatures for each strain tested. The production of extracellular DNase was inferred by the presence of a clear halo on DNase test agar around colonies of the tested strains (Palmer et al., 2012). In order to test whether planktonically grown cells were also able to secrete DNases, strains were also grown in liquid culture in triplicate. Cell-free supernatant (100 μl) of overnight liquid culture (marine broth) was then applied to wells in the DNase test agar, to verify the extracellular activity of the supernatants. Further confirmation of DNase activity was also confirmed by DNA digestion and gel electrophoresis for strains that showed high DNase activity in the filtered supernatant assays.

### Measurement of DNase Activity of Seawater

Seawater (100 μl) obtained from the North Sea was used to inoculate 5 mm agar wells of DNase test agar (as above). Any presence of a clear halo was taken to indicate DNA degrading activity. In addition, gel electrophoresis was used to examine the presence of DNA degradation by non-autoclaved and autoclaved seawater.

### DNA Extraction and 16S rRNA Gene Sequencing

Total DNA of bacterial isolates was extracted by Invitrogen Purelink^®^ Genomic DNA Mini Kit (according to the manufacturer’s protocol). The 16S rRNA gene was then amplified using the universal 16S rRNA 27F (5′-AGA GTT TGA TCC TGG CTC AG-3′) and 1492R (5′-ACG GCT ACC TTG TTA CGA CTT-3′) primers (Eurogentec^1^) with MyTaq^TM^Red Mix 2X (Bioline^2^). PCR cycles used an initial denaturation step at 95°C for 1 min, followed by 35 cycles of 15 s at 95°C, 15 s at 55°C, and 10 s extension at 72°C.

### Sequence Analysis and Tentative Identification of Bacterial Isolates With DNase Activity

The extracted DNA from each isolate was amplified and sequenced at Geneius Labs, Newcastle upon Tyne, United Kingdom^3^. Sequences were edited and aligned using BioEdit^4^ and submitted to BLAST (NCBI, United States) for identification. Consensus sequences were generated using the DNABASER tool and compared with the sequences in the Ribosomal database 2 (RDB-II). Phylogenetic analysis was performed using MEGA version 6 (Tamura et al., 2013) after obtaining the multiple alignments of data available from public databases by ClustalX 1.83. Phylogenetic trees were generated using a neighbor-joining method with a bootstrap score of 1000 replicates.

### Generating Datasets Using Bioinformatics Resources and Sequence Databases

Sequence-based screening was applied for gene discovery of extracellular DNase homologs. A function-specific dataset for the DNase gene(s) of interest was constructed for sequence-based gene analysis. The databases used to generate these datasets are the Enzyme database (Bairoch, 2000), KEGG database (Zhang and Wiemann, 2009), and the UNIPROT protein database (Apweiler et al., 2004). Metagenomic datasets and finished bacterial genomic datasets were available through the IMG/M database (Markowitz et al., 2012). By searching through the list of “genes with extracellular DNases” from every metagenome on the IMG/M database, extracellular DNase homologs (≥99%) were retrieved. The purpose of this was to gain a better insight into the diversity and representation of putative DNases in bacterial metagenomes and genomes. The databases of translated sequences from 43 environmental metagenome projects and 2136 bacterial genomes were used.

### Extracellular DNase Diversity and Functional Analysis Using Bioinformatics Tools

Datasets of generated microbial genes which showed characteristics of diverse DNase activity and produced secreted proteins were investigated further through BLAST2GO (Gotz et al., 2008). Each of the retrieved genes was used to perform BLAST-searches and sequences having higher local similarity against the sequences of homologs (if similarity exceeded 99%), were defined as secreted DNases. The taxonomic distribution and diversity analysis was carried out using MEtaGenome ANalyzer, MEGAN 5 (Huson et al., 2011). Within MEGAN, a SEED-based functional analysis of the gene reads was applied to define specific processes associated with the extracellular DNases.

## Results

### Isolation of Marine Bacteria From Diverse Habitats

**FIGURE 1:**
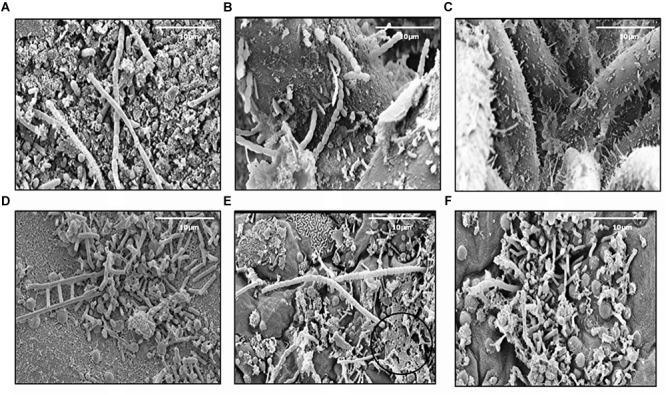
Scanning electron micrographs of the *Fucus vesiculosus* **(A–C)** and *Palmaria palmata* **(D–F)** seaweed surfaces. Micrographs **(A,D)** showing holdfast, **(B,E)** – apical tips, **(C,F)** – growth nodes. EPS like material can also be observed **(E)**, circled ×100 magnification.

In this study, 345 strains of bacteria were isolated from different marine sources and locations: seawater, biofilm, MGP (marine aggregates), seaweed and sediments (Table 1). The majority (45%) of isolates were obtained from Mid-Atlantic Ridge sediment. Diverse epiphytic microorganisms, growing on the surface of the holdfast, apical tip and growth nodes of *Fucus vesiculosus* and *Palmaria palmata*, were observed using scanning electron microscopy (Figure 1). The numbers of psychrophilic and thermophilic isolates recovered from deep-sea sediments were higher than from seaweed surfaces, seawater and MGP, while mesophilic bacteria accounted for 70% of the total isolates (Table 1).

**Table 1:** Total number of bacterial strains isolated from various environments cultivated at different temperatures.

Strain origin	Growth temperature (°C)					
	15	25	35	45	>50	Total
Mid-Atlantic Ridge sea sediment^a^	59	40	30	23	2	154
Seaweeds^b^	11	48	32	5	-	96
North Sea coastal waters and sediments	9	53	1	–	–	63
North Sea aggregates (MGP)	–	20	–	–	–	20
Biofilm from the Sea of Oman^c^	–	12	–	–	–	12
Total	79	173	63	28	2	345

*^*a*^Sea sediment obtained from the depth of around 2400–2750 m. ^*b*^Fucus vesiculosus and Palmaria palmate. ^*c*^Rocks of intertidal zone.*

### Deoxyribonuclease (DNase) Production by Cultivable Bacterial Isolates

All 345 isolates were examined for their ability to secrete DNase (Table 1). Thirty-five percent of these displayed DNase activity and were subjected to further characterization. This included identification by 1 rRNA gene sequencing. Sequences of the identified strains are available, GenBank Accession numbers (MK599164-MK599196). Most of the identified DNase producing bacteria (Supplementary Table S1) belong to the Proteobacteria (57%) followed by Firmicutes (34%), and Actinobacteria (6%) (Table 2 and Supplementary Figure S2). Most of the isolates belonging to the genera *Bacillus* (28%), *Pseudoalteromonas* (19%), and *Vibrio* (12%) produced DNases. DNase production by the genera *Terrabacter, Ochrobactrum, Rhodococcus, Arthrobacter, Planococcus*, and *Paenisporosarcina* is reported for the first time in this study. Furthermore, eight isolates from MGP showed DNase activity: *Bacillus cereus, Pseudoalteromonas espejiana, Vibrio atlanticus, Shewanella fidelis, Pseudoalteromonas citrea, Vibrio atlanticus, Cobetia amphilecti, and Pseudoalteromonas issachenkonii*.

**Table 2:** Taxonomic classification of isolated bacterial strains based on the abilities to produce DNase enzymes.

Genera	Phylum	Psychrophiles	Mesophiles	Thermophiles	Total
*Bacillus*	Firmicutes	5	7	23	35
*Lactobacillus*	Firmicutes	–	1	–	1
*Vibrio*	Proteobacteria	2	13	–	15
*Marinomonas*	Proteobacteria	1	1	–	2
*Pseudomonas*	Proteobacteria	–	7	–	7
*Pseudoalteromonas*	Proteobacteria	11	12	–	23
*Serratia*	Proteobacteria	1	3	–	4
*Exiguobacterium*	Firmicutes	2	–	–	2
*Halomonas*	Proteobacteria	1	–	–	1
*Planococcus*	Firmicutes	2	1	–	3
*Paenisporosarcina*	Firmicutes	–	1	–	1
*Idiomarina*	Proteobacteria	1	–	–	1
*Arthrobacter*	Actinobacteria	–	2	–	2
*Shewanella*	Proteobacteria	2	7	–	9
*Streptomyces*	Actinobacteria	–	3	–	3
*Microbacterium*	Actinobacteria	–	1	–	1
*Terrabacter*	Actinobacteria	–	1	–	1
*Ochrobactrum*	Proteobacteria	–	2	–	2
*Rhodococcucs*	Actinobacteria	–	1	–	1
*Rahnella*	Proteobacteria	–	1	–	1
Non-identified	N/A	5	3	–	8
Total		33	67	23	123

**FIGURE 2:**
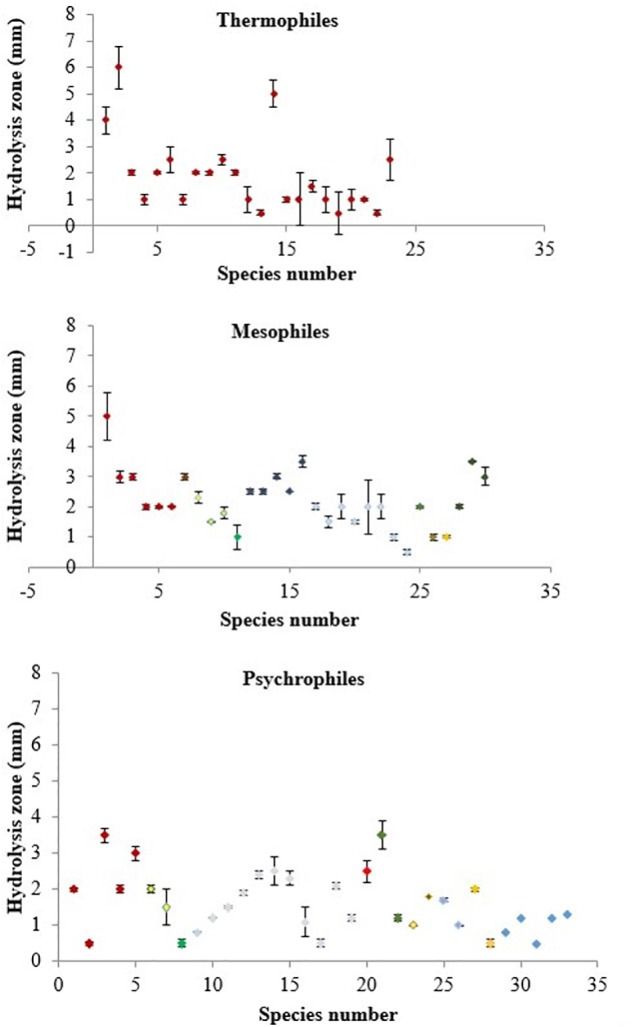
DNase activity in culture supernatants of thermophilic, mesophilic and psychrophilic isolates measured by the DNA hydrolysis zone (mm) produced on DNase Test Agar. Color coding represents each bacterial genera as follows: *Bacillus* – dark red, *Vibrio* – yellow, *Marinomonas* – green, *Pseudomonas* – dark blue, *Pseudoalteromonas* – light blue, *Planococcus, Planomicrobium, Paenisporosarcina* – orange, *Serratia* – bright red, *Arthrobacter* – purple, *Streptomyces* – brown, *Halomonas* – light purple, *Idiomarina* – dark purple, *Shewanella* – light blue, *Exiguobacterium* – light orange and non-identified – blue.

The numbers of psychrophiles, mesophiles and thermophiles belonging to each bacterial genera producing DNase are summarized in Table 2. Mesophile strains account for 54% of total DNase producing bacteria. The zone of hydrolysis for DNA degradation of the psychrophilic, mesophilic and thermophilic isolates is shown in Figure 2. Based on quantitative DNase assay, thermophilic and mesophilic bacterial isolates showed higher DNase activity than psychrophilic producers. We also observed no detectable DNAse activity in seawater samples (data not shown).

### Sequence Driven Identification, Diversity, and Distribution of Microbial Extracellular Nuclease-Like Genes in the Genome and Metagenome Databases

To explore the diversity of putative nucleases a comprehensive bioinformatics search of 43 environmental metagenomes and 2136 bacterial genomes was conducted (Supplementary Figure S1A). As a preliminary study, nuclease-like genes were surveyed across prokaryotic and eukaryotic organisms (Supplementary Table S2). In total, seven microbial genes, which showed distinctive diverse nuclease activity and produced secreted protein, were chosen as a reference for further search. These were: NucB, a sporulation-specific extracellular nuclease from *Bacillus subtilis*; NucH, a thermonuclease from *Staphylococcus aureus*; Endo I, a periplasmic nuclease from *Escherichia coli*; NucA, a DNA/RNA non-specific nuclease from *Serratia marcescens;* S1P1 nuc, an extracellular nuclease from *Penicillium melinii; Aspergillus oryzae*, Dns, an extracellular deoxyribonucleases from *Aeromonas hydrophila;* and End A, a DNA entry nuclease from *Streptococcus pneumonia*.

This set of seven identified genes were BLAST-searched against databases of draft and completed bacterial genomes and metagenomes, which produced 538 microbial nuclease-like gene reads. An additional search for putative extracellular nucleases identified a further 324 microbial nuclease-like gene reads. Each nuclease-like gene retrieved the 20 highest hits of similar gene sequences through BLAST2GO (Supplementary Table S2).

**FIGURE 3:**
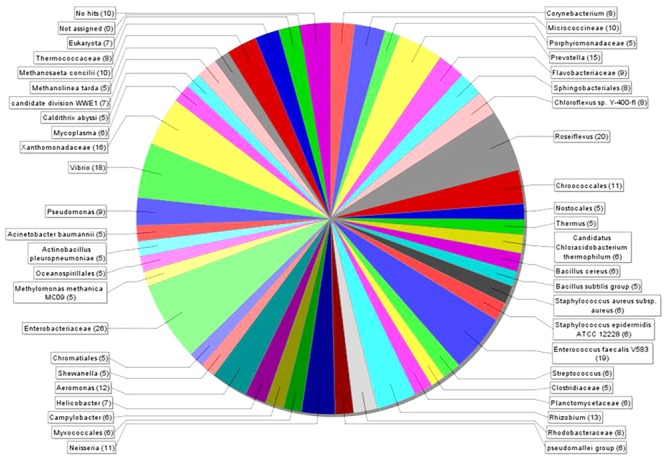
The taxonomic breakdown of microbial diversity analyzed with MEGAN 5. Visualization of the different taxonomic groups encoding 862 DNase-like genes in the bacterial genome and metagenome datasets. Each color represents individual taxonomic groups in the NCBI taxonomy and the number in the brackets denote the number of gene reads assigned to them.

MEGAN 5 was applied to estimate the microbial diversity and explore the taxonomic content of generated data. This analysis revealed 39 different taxonomic groups (Figure 3), covering 10 bacterial phyla (Supplementary Figure S1B). The highest number of putative nuclease genes was identified in *Enterobacteriaceae* [26] and *Roseiflexus* [20], followed by *Enterococcus faecalis* V583 [19], *Vibrio* [18], *Xanthomonadaceae* [16], and *Prevotella* [15] (Figure 3). The summarized phylum-level analysis (see Supplementary Figure S1A) shows that of a total 862 reads, 87% [751] were assigned to bacterial groups, with a majority of reads assigned to Proteobacteria 33% [248] and Firmicutes 25% [188]. Sequences from Archaea and Eukaryota corresponded only to 4.3 and 0.8% of the total sequences, respectively.

**FIGURE 4:**
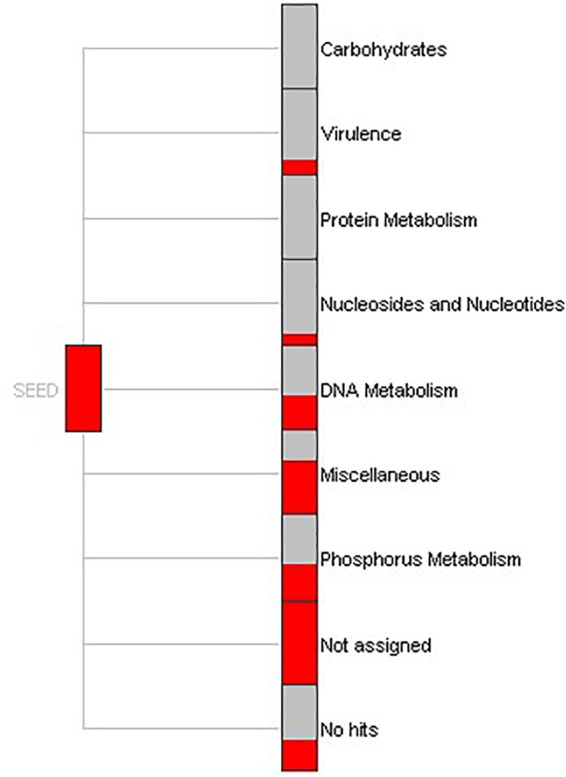
SEED-based functional assignment. Part of a SEED-based functional analysis of 862 DNase-like gene reads obtained from bacterial genome and metagenome. Each item represents a functional role in the SEED and the size of each red colored box is scaled logarithmically to represent the number of reads assigned to this.

To understand the biological processes involving the genes identified in this study, function-based screening approaches were applied. Within MEGAN, a SEED-based functional analysis of the gene reads defined the specific processes in which extracellular nucleases are implicated (Figure 4). Results revealed that a large portion of functions were expressed for DNA metabolism, phosphorus metabolism and virulence. The biological functions were similar to the functions elucidated by gene mapping performed in BLAST2GO. Noticeably, the nucleic acid phosphodiester bond hydrolysis and sporulation resulting in the formation of a cellular spore showed the greatest annotation score (Supplementary Figure S3). Nevertheless, outcomes (see Supplementary Figure S4) suggest additional important processes involving nucleases, including metabolism of nitrogen, phosphorous, purine, small molecules like tRNA, and response to stress stimulus.

## Discussion

Bacterial extracellular enzymes are ubiquitous in the ocean and play an important role in the cycling and fate of organic matter. However, knowledge regarding DNase diversity in marine bacteria in general, and in marine aggregates more specifically, remains rudimentary. DNase production has been reported in several bacterial species, including *Serratia marcescens* (Nestle and Roberts, 1969), marine isolates of *Vibrio* sp. (Maeda and Taga, 1976), *Pseudomonas aeruginosa* (Mulcahy et al., 2010), *Myroides, Planococcus, Sporosarcina* and *Halomonas* (Dang et al., 2009), and *Bacillus licheniformis* (Nijland et al., 2010). In addition, there are some studies on DNases produced by the human pathogen *Streptococcus* (Porschen and Sonntag, 1974). However, most studies have focused only on individual cultivable species. There are no studies on the overall diversity of DNases produced by bacteria in general or by marine aggregate associated bacteria. In this study we used a culture based approach and bioinformatics tools together, to highlight the diversity of marine bacterial production of nucleases and DNases.

### Deoxyribonuclease Production by Cultivable Marine Bacteria

In our current study, bacteria were isolated from various environments (seaweed surfaces, North Sea waters, mid-Atlantic Ridge sediments and coastal waters of Oman) (Table 1) and are of different types (free-living or attached) and from a range of temperatures (15–45^o^C). For seaweed associated bacteria, an EPS matrix was visualized on the seaweed surface by ESM circled in Figure 1E. This layer is presumably secreted by the algae and the commensal epibiotic bacteria on the seaweed surface to reduce fouling, and protection against harsh conditions and predation (Armstrong et al., 2001; Wiese et al., 2009; Goecke et al., 2010; Wahl et al., 2012). Subsequently, these macroalgae may release EPS materials into the water column, that are then colonized by bacteria and would contribute to the MGP pool in the ocean. These particles may aggregate further to form marine snow and can settle into the deep ocean through sedimentation. Exopolymer release by algae and diatoms is well evidenced (Thornton, 2004, 2014; Chen and Thornton, 2015; Li et al., 2015) but the chemical composition of these particles as they evolve and sink is not well studied. In particular, the presence of DNA in these particles is unknown. It is thought that DNA is abundant in higher concentrations in sediments compared to seawater and that 90% of the DNA found in sediments is extracellular DNA (Dell’Anno et al., 2002; Nielsen et al., 2007; Torti et al., 2015). This suggests that this DNA is transported into the deep sea by sinking particles.

In this work, 35% of all the cultivable bacteria obtained produced extracellular DNase. The production of DNase seems to be common among a diverse array of marine bacterial isolates and is greater in mesophilic bacteria (54%) than in psychrophilic or thermophilic as our results demonstrated (Table 2 and Figure 2). A possible explanation is that marine bacterial DNase may be more active at mesophile conditions of 20–45^o^C (Ahrenholtz et al., 1994). Most of the cultivable bacteria that produced DNase in this study belong to 20 genera, affiliated to Proteobacteria (53%) and Firmicutes (34%) (Supplementary Figure S2). It has been observed that Proteobacteria employ nuclease based bacteriocins (NBs) as a defense mechanism (Parret and De Mot, 2002; Sharp et al., 2017).

Most of the isolates that were able to produce DNase belonged to the genus *Bacillus* (28%) and all were identified as thermophiles. *Bacillus* are well-known enzyme producers (Ferrero et al., 1996; Lei et al., 2017; Parab et al., 2017; Anjum et al., 2018) including nuclease production, for example, NucB production by *Bacillus licheniformis* (Nijland et al., 2010). The next highest cultivable genus was *Pseudoalteromonas* (18%), which species of which are known for their production of bioactive compounds and enzymes (Tutino et al., 2002; Zeng et al., 2006; de Pascale et al., 2008; Bian et al., 2012; Bhattacharya et al., 2018). A recent study of extracellular enzymes from marine cultivable bacteria from The New Britain Trench showed that *Pseudoalteromonas* produced various extracellular enzymes, mostly proteases and chitinases (García-Fraga et al., 2015; Liu et al., 2018). Another study on cultivable bacteria in Laizhou Bay, China, showed that *Pseudoalteromonas* was the most common cultivable genus producing extracellular proteases (Li et al., 2017). Moreover, *Pseudoalteromonas* spp. have also been reported to produce nuclease baceriocins (Desriac et al., 2010).

Here, bacteria associated with marine aggregates were investigated for nuclease production for the first time. Our results revealed that eight out of 20 isolates from MGP were able to secrete nucleases. These isolates belong to the families Pseudoalteromonadacea (Azam and Malfatti, 2007), Vibrionaceae (Azam, 1998), Bacillaceae (Salazar and Sunagawa, 2017), Shewanellaceae (Salazar and Sunagawa, 2017), and Halomonadaceae (Salazar and Sunagawa, 2017) most of which have been reported previously (Dang et al., 2009; Balabanova et al., 2017). Although extracellular enzymes from *Vibrio* (Bunpa et al., 2016) and extracellular agents and enzymes from *Pseudoalteromonas citrea* have been reported previously (Holmström and Kjelleberg, 1999; Roca et al., 2016; Yamada et al., 2016; Imbs et al., 2018), extracellular DNases from bacteria isolated specifically from MGPs has not been described previously. In addition, there is evidence that *Pseudomonas* and *Vibrio* produce extracellular deoxyribonucleases to utilize DNA as a nutrient source as well as synthesizing DNases for horizontal gene transfer of DNA as part of natural transformation (Blokesch and Schoolnik, 2008; Mulcahy et al., 2010). However, the observation that *Vibrio atlanticus* and *Psudoalteromonas citrea* isolated from MGP can produce DNase, is interesting as it may implicate DNase in the dispersal of MGP, based on our hypothesis that eDNA is a key component of MGPs and plays a role in their physical integrity.

Extracellular enzymes produced by heterotrophic bacteria are key players in organic matter cycling in the ocean (Azam and Malfatti, 2007; Rier et al., 2014; Balmonte et al., 2016; Burns et al., 2016) and bacterial extracellular enzyme activity in aggregates is reported to be two orders of magnitude higher than in free-living bacteria (Smith et al., 1992; Grossart et al., 2006; Ziervogel et al., 2010; Kellogg and Deming, 2014). These extracellular enzymes are thought to be involved in the dissolution of MGPs and marine snow (D’ambrosio et al., 2014; Balmonte et al., 2016; Luo et al., 2017; Baltar, 2018; Ivančić et al., 2018). However, the effect of nucleases, specifically has hitherto been overlooked, with regard to the implications for MGP and marine snow dispersal. We were also unable to detect DNase activity in seawater samples, indicating that the level of enzyme activity may be much lower than concentrations which might be expected to occur inside MGPs.

Additional studies are required to study the production of nucleases *in situ*. However, results with cultivable isolates revealed that DNase is expressed by various bacteria. The versatile ability of marine isolates to produce DNase suggests that they play an important ecological role in extracellular DNA degradation in the marine environment. The present study provides data on DNase production by marine cultivable bacteria from aggregates for the first time and hence provides further insights into the diversity of extracellular enzyme production by marine bacteria.

### DNase Gene Diversity a Bioinformatics Study

The MetaBIOME database was employed previously to explore and identify commercially applicable enzymes from metagenomes (Sharma et al., 2010; Sharma and Taylor, 2015) and as an advanced tool for exploring novel genes involved in marine microbial metabolism (Kennedy et al., 2008, Kennedy et al., 2010). Similarly, in this study, the IMG/M data management and analysis system was explored for nucleases. The IMG/M database comprises both microbial whole genomes and metagenomes. As a simple, yet comprehensive analysis tool to search for currently available genes of interest, it has advantages over other applied databases, for example, GenDB/JCoast (Richter et al., 2008) and Magnifying Genome/MicroScope (Vallenet et al., 2013).

A bioinformatics survey of 43 environmental metagenomes and 2136 bacterial genomes, together with taxonomic analysis, revealed a high diversity of microbial extracellular nuclease-like genes. Interestingly, nuclease-like enzyme positive genotypes were found in 39 different taxonomic groups, representing one-third of bacterial phyla. Taxonomic classification indicated that the dominant bacterial phyla were Proteobacteria and Firmicutes, accounting for 33 and 25%, respectively, of the total bacterial reads, respectively (Figure 3). This reinforces our experimental data where 53% of the cultured nuclease enzyme producing bacteria belonged to the phylum Proteobacteria and 34% to Firmicutes. This concurrence in the results of the culture-based methods and the bioinformatics tools suggest that marine Proteobacteria and Firmicutes are important producers of nucleases. Furthermore, data presented in Table 2 are also in agreement with a recent study that developed a bioinformatics pipeline to identify nuclease bacteriocins (NBs) in bacteria that revealed that NBs are found commonly in gamma-proteobacteria (Sharp et al., 2017).

Microbial diversity of extracellular enzymes using an openly available sequence-based approach has been used successfully for esterases (Elend et al., 2006). However, information regarding the variety of secreted nucleases is still scant. The sequence driven analysis suggests that microbial communities are able to produce diverse extracellular nuclease-like enzymes. However, the taxonomy-based function predictions are limited, as they depend on databases that are constructed from sequences of cultured bacteria, which restricts the understanding of natural community functions.

BLAST2GO gene mapping and SEED-based functional analysis underlined the fact that DNases are also centrally important in DNA metabolism and involved in a number of intracellular DNA processing steps (Figure 4). Because bacteria can produce DNases to utilize extracellular DNA as a source of carbon, nitrogen and phosphorus we also surveyed intracellular putative DNase enzyme genes in this work. DNases also play a role in virulence, degradation of neutrophil DNA extracellular traps (NETs) and in biofilm degradation (Dubnau, 1999; Thomas and Nielsen, 2005; Vorkapic et al., 2016; Veening and Blokesch, 2017). Bacteria employ DNases to also compete with other bacteria for space or resources and thus these enzymes have an important ecological function in survival (Cao et al., 2017). Nucleases digest DNA that can be used as the sole nutrient source by biofilm-forming bacteria *Pseudomonas aeruginosa* and *Shewanella oneidensis* (Mulcahy et al., 2010; Godeke et al., 2011). Another study reported that the balance of extracellular DNA in the marine environment is largely regulated by DNase, which is important for the functioning of deep-sea ecosystems (Dell’Anno and Danovaro, 2005). Our findings regarding the role of DNases in cellular metabolism of nitrogen and phosphorus, suggest that DNase secretion may also play an important role in the fate of extracellular DNA in the natural environment, particularly with regard to the dynamics and stability of marine aggregates.

Culture-based and bioinformatics tools used to investigate the diversity of DNases allowed us to gain insight into the presence and expression of DNase in cultivable and non-cultivable marine bacteria. Additionally diverse bacterial genera that were recovered were able to produce DNases, and the function based investigation of the sequence reads facilitated an appreciation of the ecological importance of extracellular DNases. The present study provides new insights into the abundance, hidden diversity, and environmentally important functions of microbes carrying DNase-like genes.

The most notable finding to emerge from this study is that the bacterial community associated with MGPs is able to secrete extracellular DNases. This is an important element that could contribute to our understanding of extracellular enzymes present within gel particles and marine snow in the ocean. In addition, it might provide answers to the key questions surrounding what proportion of particulate organic matter sinks into the deep ocean and what regulates this process.

There is a need to further understand the diversity of MGP associated bacteria and their ability to produce extracellular DNases in the ocean. We are now investigating the effects of extracellular DNases on MGP dissolution. This can expand our knowledge of bacterial DNases and the role they play in the dynamics of MGP and consequently carbon cycling in the oceans.

## Author Contributions

JB contributed to the conception and design of the work. NR, EL, and AA-W has contributed in the acquisition, analysis and interpretation of the data. AA-W and NR wrote and drafted the manuscript. JB, SD, and RU-G critically revised the manuscript.

## Conflict of Interest Statement

The authors declare that the research was conducted in the absence of any commercial or financial relationships that could be construed as a potential conflict of interest.

## Footnotes

1 https://secure.eurogentec.com/eu-home.html

2 https://www.bioline.com/mytaq-red-mix.html

3 http://www.geneiuslabs.co.uk/

4 http://www.mbio.ncsu.edu/BioEdit/bioedit.html

## Supplementary Material

FIGURE S1 Phylogenetic diversity of the putative DNase-like enzyme producing taxonomic groups as calculated by MEGAN 5. **(A)** Phylum-level summary of MEGAN analysis. **(B)** A low level view of the MEGAN analysis. Each node represents a taxon in the NCBI taxonomy, and the size of the node is scaled logarithmically to represent the number of reads assigned directly to the taxon.

FIGURE S2 Phylogenetic diversity at phylum level of the cultured DNase enzyme producing bacteria where 53% belong to the phylum Proteobacteria, 34% Firmicutes, and 6% Actinobacteria.

FIGURE S3 A Directed Acyclic Graph (DAG) visualizing the hierarchical structure of the Gene Ontology (GO) in biological processes involving DNases resulting in sporulation. The darker the color of the node, the greater the number of BLAST hits and the higher the score values. All nodes contain the hit annotation scores in numbers.

FIGURE S4 A Directed Acyclic Graph (DAG) visualizing the biological process involving DNAses resulting in metabolism of nitrogen, phosphorous, purine, small molecules such as tRNA and response to stress stimulus. The darker the color of the node the greater the number of BLAST hits and the higher the score values. All nodes contain the hit annotation scores in numbers.

TABLE S1 List of strains used.

TABLE S2 The dataset of the BLAST2GO results of 832 DNase-like gene reads.
