# Supplementary figures and images for: Secretion of DNases by Marine Bacteria: A Culture Based and Bioinformatics Approach
Source: Front Microbiol. 2019 May 7;10:969. doi: 10.3389/fmicb.2019.00969 (PMC6514286; doi:10.3389/fmicb.2019.00969)

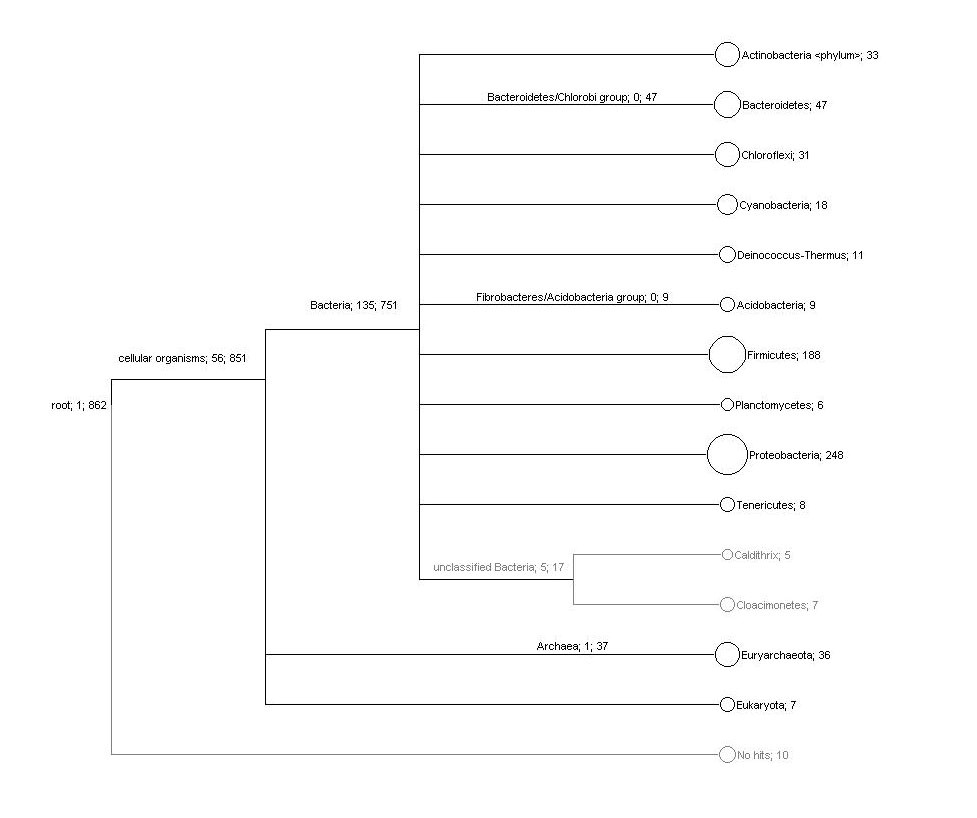

Supplement: FIGURE S1 — Phylogenetic diversity of the putative DNase-like enzyme producing taxonomic groups as calculated by MEGAN 5. (A) Phylum-level summary of MEGAN analysis. (B) A low level view of the MEGAN analysis. Each node represents a taxon in the NCBI taxonomy, and the size of the node is scaled logarithmically to represent the number of reads assigned directly to the taxon. [file Image_1.JPEG]

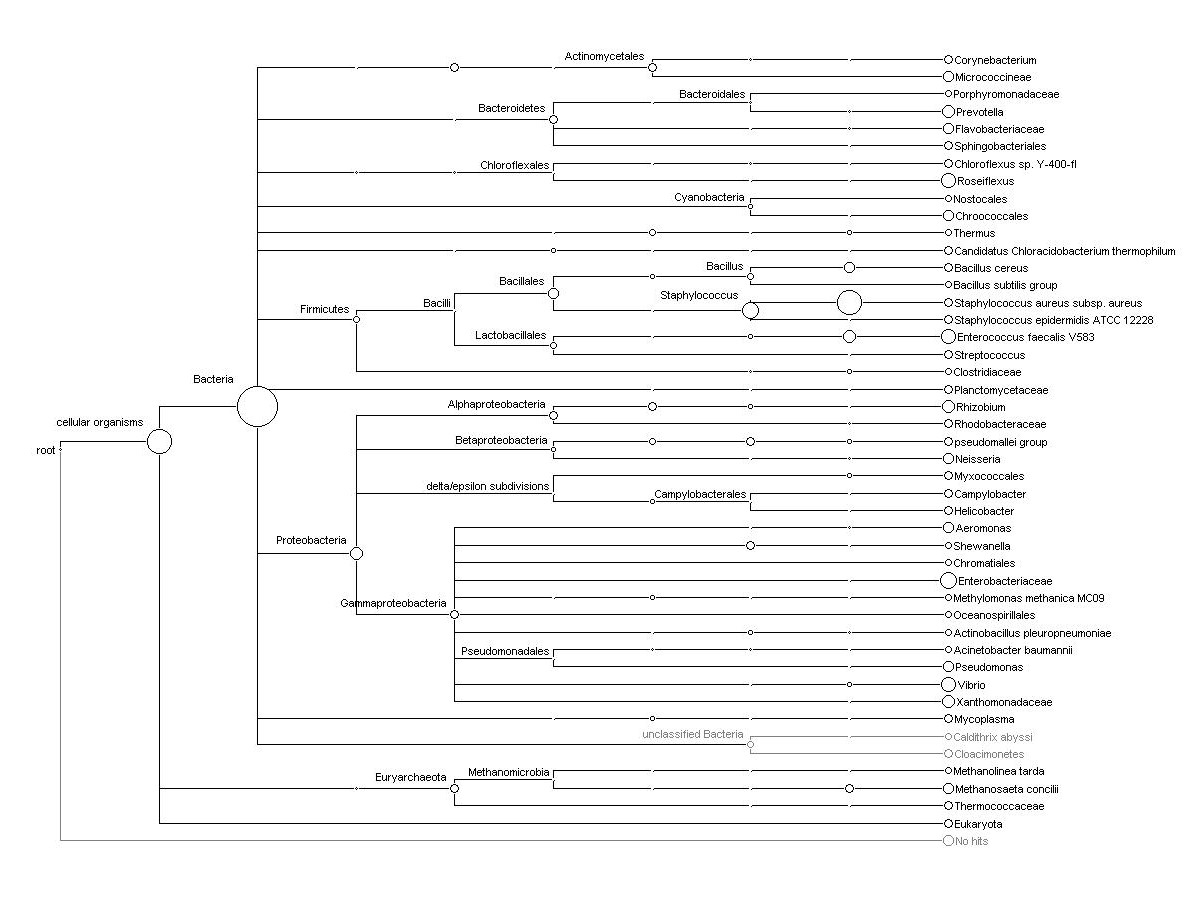

Supplement: FIGURE S2 — Phylogenetic diversity at phylum level of the cultured DNase enzyme producing bacteria where 53% belong to the phylum Proteobacteria, 34% Firmicutes, and 6% Actinobacteria. [file Image_2.JPEG]

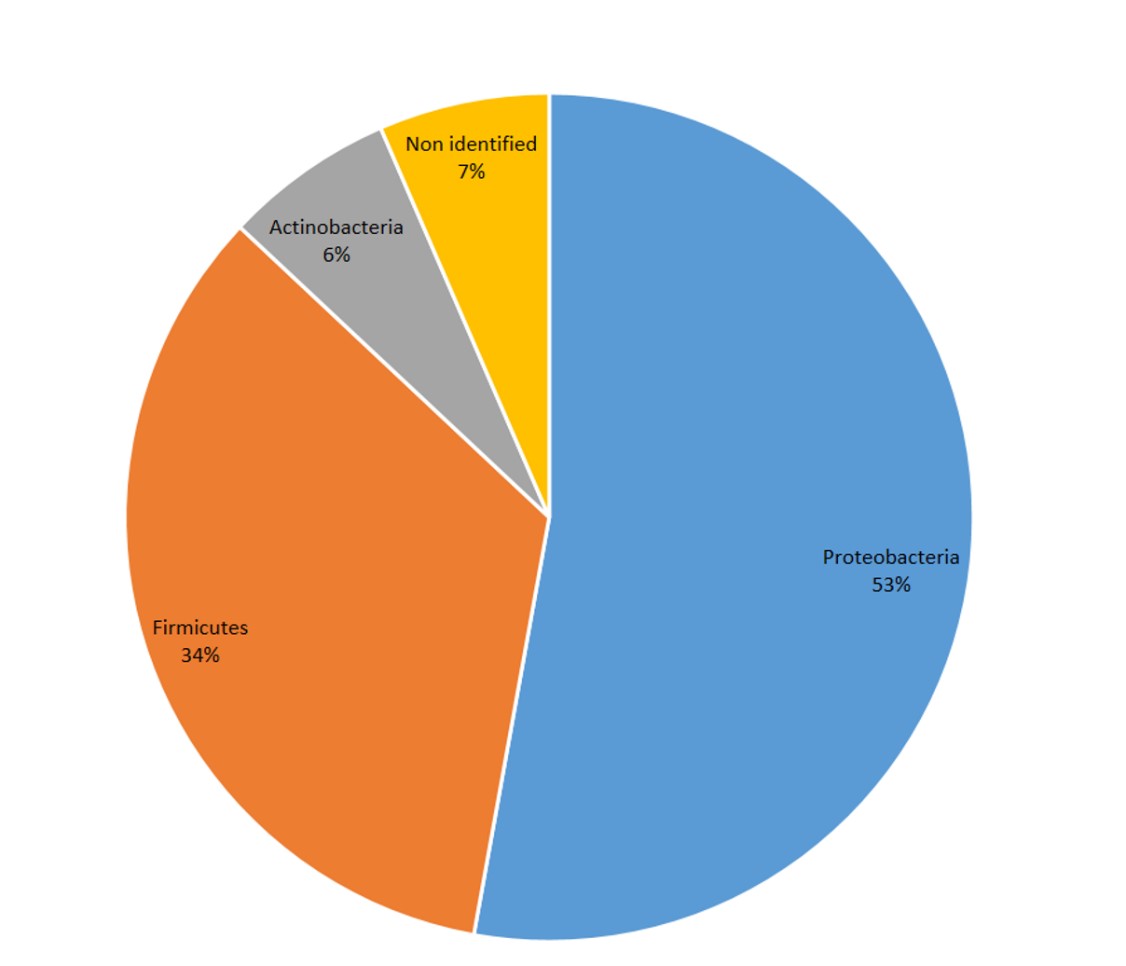

Supplement: FIGURE S3 — A Directed Acyclic Graph (DAG) visualizing the hierarchical structure of the Gene Ontology (GO) in biological processes involving DNases resulting in sporulation. The darker the color of the node, the greater the number of BLAST hits and the higher the score values. All nodes contain the hit annotation scores in numbers. [file Image_3.JPEG]

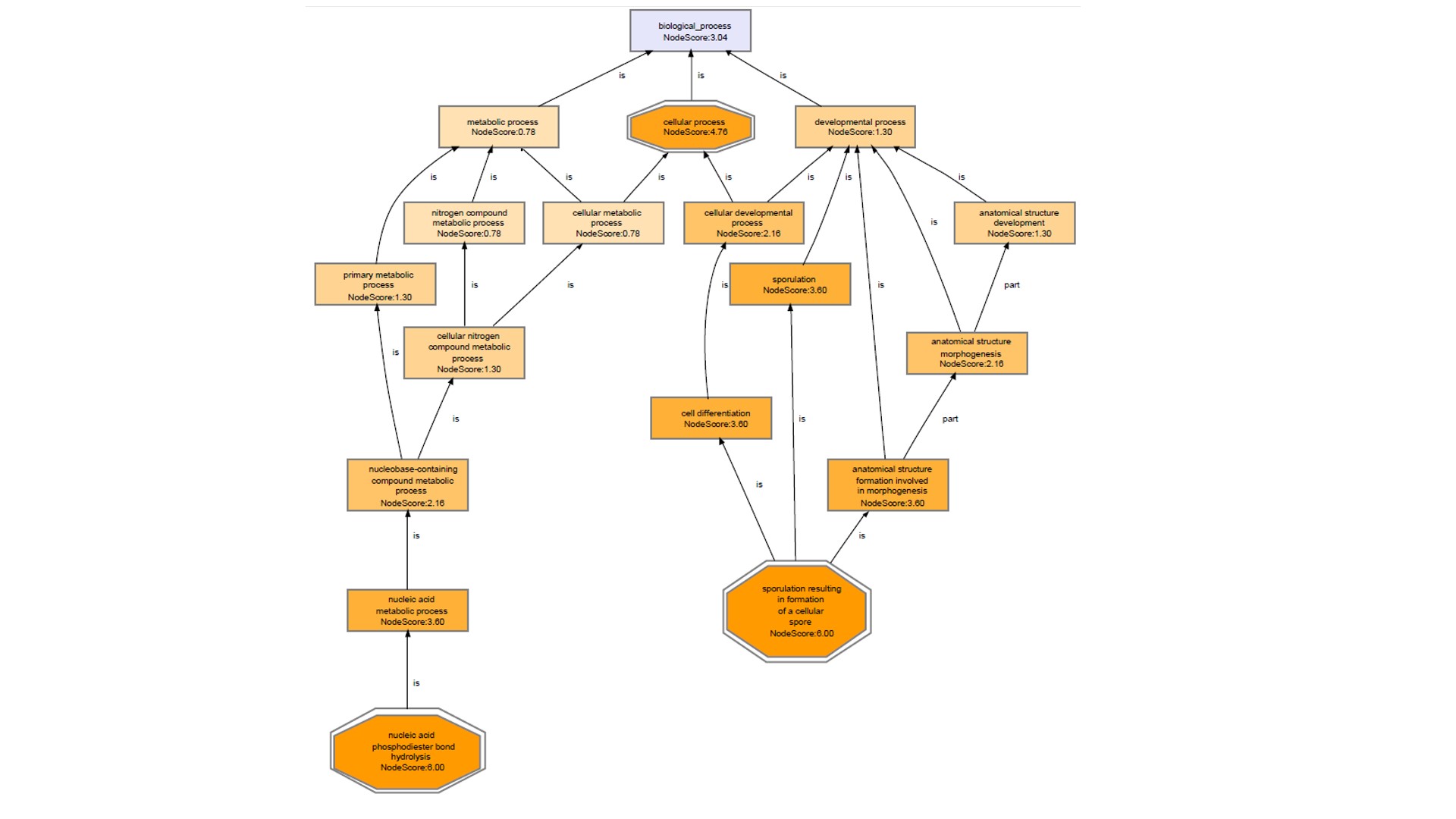

Supplement: FIGURE S4 — A Directed Acyclic Graph (DAG) visualizing the biological process involving DNAses resulting in metabolism of nitrogen, phosphorous, purine, small molecules such as tRNA and response to stress stimulus. The darker the color of the node the greater the number of BLAST hits and the higher the score values. All nodes contain the hit annotation scores in numbers. [file Image_4.JPEG]

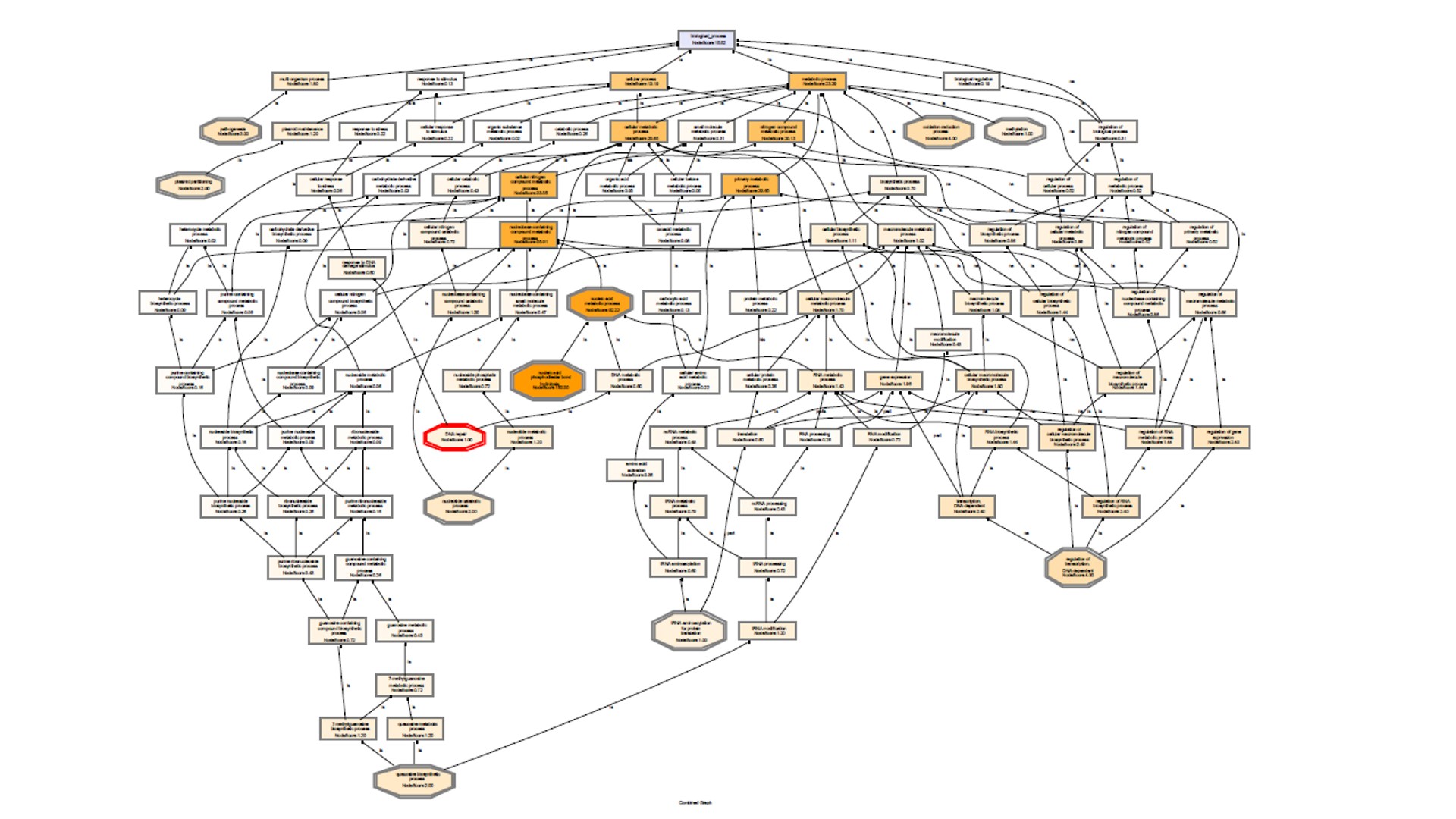

Supplement: Supplementary file 5 [file Image_5.JPEG]
