# Supplementary material for: Secretion of DNases by Marine Bacteria: A Culture Based and Bioinformatics Approach
Source: Front Microbiol. 2019 May 7;10:969. doi: 10.3389/fmicb.2019.00969 (PMC6514286; doi:10.3389/fmicb.2019.00969)
Supplement: TABLE S1 — List of strains used. [file Table_1.DOCX]

**Supplementary Table 1:**

List of 123 tentatively identified bacterial strains isolated in this work and their optimal growth temperatures.

| **Strains No.** | **Identity** | ***16S rRNA identity*** | **Sequence similarity (%)** | **Temperature (°C)** |
| --- | --- | --- | --- | --- |
| 1 | AV 072 | *Pseudoalteromonas sp. BSw20057* | 99.0 | 16 |
| 2 | AV 062 | *Pseudoalteromonas tetraodonis 14160T* | 99.5 | 16 |
| 3 | AV 025 | *Pseudoalteromonas carrageenovora ATCC 12662T* | 99.1 | 16 |
| 4 | AV 074 | *Pseudoalteromonas prydzensis MB8-11T* | 98.3 | 16 |
| 5 | AV 210 | *Pseudoalteromonas marina* | 99.8 | 16 |
| 6 | AV 681 | *Pseudoalteromonas aliena KMM 3562T* | 99.9 | 16 |
| 7 | AV 042 | *Pseudoalteromonas paragorgicola* | 99.7 | 16 |
| 8 | AV 023 | *Pseudoalteromonas nigrifaciens NCIMB 8614T* | 99.9 | 16 |
| 9 | AV 043 | *Pseudoalteromonas espejiana NCIMB 2127T* | 99.4 | 16 |
| 10 | AV 530 | *Pseudoalteromonas undina NCIMB 2128T* | 99.6 | 16 |
| 11 | AV 211 | *Pseudoalteromonas elyakovii KMM 162T* | 99.6 | 16 |
| 12 | AV 021 | *Bacillus circulans* | 99.8 | 16 |
| 13 | AV 019 | *Bacillus simplex* | 99.5 | 16 |
| 14 | AV 020 | *Bacillus simplex* | 99.7 | 16 |
| 15 | AV 022 | *Bacillus circulans* | 98.8 | 16 |
| 16 | AV 230 | *Bacillus firmus* | 99.2 | 16 |
| 17 | AV 221 | *Serratia plymuthica* | 99.8 | 16 |
| 18 | AV 013 | *Vibrio cyclitrophicus* | 99.8 | 16 |
| 19 | AV 014 | *Vibrio splendidus* | 99.9 | 16 |
| 20 | AV 009 | *Marinomonas primoryensis* | 99.5 | 16 |
| 21 | AV 041 | *Planococcus donghaensis* | 98.6 | 16 |
| 22 | AV 717 | *Planococcus maritimus* | 98.0 | 16 |
| 23 | AV 003 | *Exiguobacterium undae* | 98.6 | 16 |
| 24 | AV 017 | *Exiguobacterium marinum* | 99.0 | 16 |
| 25 | AV 026 | *Halomonas alkaliphila* | 99.9 | 16 |
| 26 | AV 027 | *Idiomarina loihiensis* | 99.9 | 16 |
| 27 | AV 441 | *Shewanella olleyana* | 97.0 | 16 |
| 28 | AV 323 | *Shewanella japonica KMM 3299T* | 99.4 | 16 |
| 29 | AV 415 | *Unknown* | unidentified | 16 |
| 30 | AV 417 | *Unknown* | unidentified | 16 |
| 31 | AV 891 | *Unknown* | unidentified | 16 |
| 32 | AV 892 | *Unknown* | unidentified | 16 |
| 33 | AV 893 | *Unknown* | unidentified | 16 |
| 34 | AV 005 | *Bacillus cereus* | 98.8 | 37 |
| 35 | Natto | *Bacillus subtilis natto* | 99.5 | 37 |
| 36 | AV 854 | *Bacillus pumilus* | 99.5 | 37 |
| 37 | AV 850 | *Bacillus licheniformis* | 99.2 | 37 |
| 38 | AV 045 | *Bacillus mycoides* | 99.2 | 28 |
| 39 | AV 039 | *Bacillus baekryungensis* | 98.0 | 28 |
| 40 | AV 040 | *Lactobacillus plantarum* | 99.3 | 37 |
| 41 | AV 001 | *Pseudomonas stutzeri* | 99.8 | 37 |
| 42 | AV 011 | *Pseudomonas synxantha* | 99.7 | 37 |
| 43 | AV 012 | *Pseudomonas oleovorans* | 99.2 | 37 |
| 44 | AV 236 | *Pseudomonas fulva* | 98.9 | 37 |
| 45 | AV 024 | *Pseudomonas tolassi* | 99.4 | 37 |
| 46 | AV 028 | *Vibrio pomeroyi* | 98.0 | 37 |
| 47 | AV 291 | *Vibrio gigantis* | 98.9 | 37 |
| 48 | AV 303 | *Vibrio nitriegens* | 97.0 | 37 |
| 49 | AV 037 | *Paenisporosarcina quisquiliarum* | 98.6 | 28 |
| 50 | AV 159 | *Pseudoalteromonas issanchenkonii* | 99.9 | 28 |
| 51 | AV 164 | *Pseudoalteromonas aliena* | 99.9 | 28 |
| 52 | AV 682 | *Pseudoalteromonas carrageenora* | 99.9 | 28 |
| 53 | AV 184 | *Pseudoalteromonas arctica* | 99.9 | 28 |
| 54 | AV 142 | *Pseudoalteromonas prydzensis* | 99.5 | 28 |
| 55 | AV 152 | *Pseudoalteromonas mariniglutinosa* | 100.0 | 28 |
| 56 | AV 606 | *Pseudoalteromonas undina* | 100.0 | 28 |
| 57 | AV 007 | *Pseudoalteromonas mariniglutinosa* | 98.9 | 28 |
| 58 | AV 056 | *Marinomonas arenicola KMM 3893T* | 99.7 | 28 |
| 59 | AV 002 | *Planococcus sp.* | 98.0 | 28 |
| 60 | AV 319 | *Arthrobacter agilis DSM 20550T* | 99.2 | 28 |
| 61 | AV 283 | *Streptomyces griseus* | 99.9 | 28 |
| 62 | AV 284 | *Streptomyces rimosus* | 99.9 | 28 |
| 63 | AV 285 | *Streptomyces sp.* | 99.0 | 28 |
| 64 | AV 111 | *Bacillus licheniformis* | 98.9 | 45 |
| 65 | AV 115 | *Bacillus licheniformis* | 99.3 | 45 |
| 66 | AV 145 | *Bacillus pumilus* | 99.5 | 45 |
| 67 | AV 118 | *Bacillus licheniformis* | 99.2 | 45 |
| 68 | AV 119 | *Bacillus licheniformis* | 99.6 | 45 |
| 69 | AV 226 | *Bacillus licheniformis* | 99.5 | 45 |
| 70 | AV 229 | *Bacillus tequilensis* | 99.9 | 45 |
| 71 | AV 233 | *Bacillus vallismortis* | 99.0 | 45 |
| 72 | AV 276 | *Bacillus pumilus* | 99.3 | 45 |
| 73 | AV 228 | *Bacillus sonorensis* | 96.5 | 45 |
| 74 | AV 231 | *Bacillus amyloliquefaciens* | 99.0 | 45 |
| 75 | AV 234 | *Bacillus licheniformis* | 99.4 | 45 |
| 76 | AV 238 | *Bacillus sp.* | 98.0 | 45 |
| 77 | AV 239 | *Bacillus licheniformis* | 98.0 | 45 |
| 78 | AV 240 | *Bacillus sonorensis* | 99.3 | 45 |
| 79 | AV 278 | *Bacillus decolorationis* | 96.0 | 45 |
| 80 | AV 270 | *Bacillus pseudofirmus* | 99.2 | 45 |
| 81 | AV 254 | *Bacillus safensis* | 99.6 | 45 |
| 82 | AV 265 | *Bacillus alveayuensis* | 98.0 | 45 |
| 83 | AV 271 | *Bacillus hwajinpoensis SW-72T* | 99.8 | 45 |
| 84 | AV 245 | *Bacillus firmus* | 97.0 | 45 |
| 85 | AV 225 | *Bacillus safensis* | 97.0 | 45 |
| 86 | AV 279 | *Bacillus tequilensis* | 98.0 | 45 |
| 87 | TR4A | *Shewanella putrefaciens* | 98.36 | 28 |
| 88 | AW1 | *Vibrio splendidus* | 95 | 28 |
| 89 | AW2 | *Serratia marcescens* WW4 | 100 | 28 |
| 90 | AW3 | *Serratia marcescens* WW4 | 96 | 28 |
| 91 | AW4 | *Shewanella putrefaciens* Hammer 95 | 91 | 28 |
| 92 | AW5 | *Shewanella putrefaciens* | 98 | 28 |
| 93 | AW8 | Un identified | *NA* | 28 |
| 94 | AW101 | Un identified | *NA* | 28 |
| 95 | AW104 | Un identified | *NA* | 28 |
| 96 | LB1 | *Vibrio alginolyticus* | 99 | 28 |
| 97 | LB3 | *Vibrio pelagius* | 97 | 28 |
| 98 | NB11 | *Vibrio parahaemolyticus* | 97 | 28 |
| 99 | NB21 | *Vibrio parahaemolyticus* | 97 | 28 |
| 100 | MB11 | *Vibrio harveyi* | 96 | 28 |
| 101 | MB12 | *Vibrio alginolyticus* | 98 | 28 |
| 102 | MB12Y | *Pseudoalteromonas piscicida* | 99 | 28 |
| 103 | MB44 | *Vibrio alginolyticus* | 97 | 28 |
| 104 | A31 | *Pseudoalteromonas citrea* | 99 | 28 |
| 105 | D2 | *Halocynthiibacter namhaensis* | 97 | 28 |
| 106 | E2 | *Bacillus cereus* | 99 | 28 |
| 107 | F3 | *Pseudoalteromonas espejiana* | 99 | 28 |
| 108 | G22 | *Vibrio atlanticus* | 99 | 28 |
| 109 | H2 | *Shewanella fidelis* | 99 | 28 |
| 110 | I41 | *Pseudovibrio ascidiaceicola* | 99 | 28 |
| 111 | L3 | *Pseudoalteromonas citrea* | 99 | 28 |
| 112 | M2 | *Microbulbifer hydrolyticus* | 99 | 28 |
| 113 | O2 | *Polaribacter atrinae* | 98 | 28 |
| 114 | P21 | *Dermacoccus nishinomiyaensis* | 99 | 28 |
| 115 | P22 | *Micrococcus yunnanensis* | 99 | 28 |
| 116 | Q2 | *Psychrobacter cryohalolentis* | 99 | 28 |
| 117 | S2 | *Psychrobacter aquaticus* | 86 | 28 |
| 118 | T2 | *Psychrobacter maritimus* | 98 | 28 |
| 119 | U2 | *Vibrio atlanticus* | 99 | 28 |
| 120 | V2 | *Cobetia amphilecti* | 99 | 28 |
| 121 | W2 | *Shewanella surugensis* | 98 | 28 |
| 122 | X2 | *Pseudoalteromonas issachenkonii* | 99 | 28 |
| 123 | Y | *Polaribacter atrinae* | 98 | 28 |
