# Supplementary material for: Secretion of DNases by Marine Bacteria: A Culture Based and Bioinformatics Approach
Source: Front Microbiol. 2019 May 7;10:969. doi: 10.3389/fmicb.2019.00969 (PMC6514286; doi:10.3389/fmicb.2019.00969)
Supplement: TABLE S2 — The dataset of the BLAST2GO results of 832 DNase-like gene reads. [file Table_2.DOCX]

**Supplementary Table 1:**

List of predicted nucleases among sequenced prokaryotic and eukaryotic organisms, based on the secreted (+), intracellular (-) or not defined (ND) subcellular location, collected from UniProtKB database. Data presented as secreted (+) or intercellular (-) indicates that there is experimental evidence for the existence of protein. When the existence of protein is unsure the data are presented as not defined (ND).

| **Kingdom** | **Phylum** | | **Organism** | **Nuclease / protein name** | **Gene/locus name** | **aa seq** | **Location** |
| --- | --- | --- | --- | --- | --- | --- | --- |
| **Bacteria** | Acidobacteria | | *Acidobacteria bacterium* (Ellin345) | Nuclease-related protein | Acid345_4695 | 206 | ND |
|  |  | | *Solibacter usitatus* ( Ellin6076) | S1/P1 nuclease | Acid_5845 | 261 | ND |
|  | Actinobacteria | | *Corynebacterium glutamicum* | Predicted extracellular nuclease | nuc Cgl2592 cg2868 | 916 | ND |
|  |  | | *Eggerthella lenta* ATCC 25559 | HNH endonuclease | Elen_0844 | 127 | ND |
|  |  | | *Micrococcus sedentarius* | Predicted extracellular nuclease | Ksed_15630 | 802 | ND |
|  |  | | *Slackia heliotrinireducens* ATCC 29202 | HNH endonuclease | Shel_08210 | 97 | ND |
|  |  | | *Streptomyces antibioticus* | 34KDa extracellular nuclease | N/A | 9 | + |
|  |  | | *Streptosporangium roseum* | Extracellular nuclease like protein | Sros_3284 | 795 | ND |
|  | Proteobacteria | | *Caulobacter crescentus* | Nuclease, putative | CC_0544 | 695 | ND |
|  |  | | *Citreicella sp.* SE45 | Extracellular nuclease | CSE45_0773 | 1183 | - |
|  |  | | *Magnetospirillum magneticum (strain* AMB-1 / ATCC 700264) | Nuclease | amb4452 | 339 | ND |
|  |  | | *Oceanicola batsensis* HTCC2597 | Extracellular nuclease | OB2597_06605 | 1215 | ND |
|  |  | | *Roseovarius sp.* 217 | Extracellular nuclease | ROS217_14766 | 1682 | - |
|  |  |  | *Sagittula stellata* E-37 | Extracellular nuclease | SSE37_13468 | 1407 | - |
|  |  |  | *Burkholderia mallei* ATCC 23344 | Extracellular nuclease, putative | BMAA0259 | 624 | ND |
|  |  | | *Aromatoleum aromaticum* | Putative DNA/RNA NON-specific endonuclease | Nuc1 | 256 | ND |
|  |  |  | *Burkholderia mallei* ATCC 23344 | Endonuclease | Nuc BMA1476 | 207 | ND |
|  |  |  | *Anaeromyxobacter dehalogenans* | HNH nuclease | Adeh_0859 | 370 | ND |
|  |  | | *Geobacter metallireducens* DSM 7210 | HNH endonuclease | Gmet_0936 | 104 | ND |
|  |  | | *Sorangium cellulosum* | Putative nuclease | sce7025 | 233 | ND |
|  |  | | *Syntrophobacter fumaroxidans* DSM 10017 | Nuclease (SNase domain protein) | Sfum_3457 | 222 | ND |
|  |  | | *Syntrophobacter fumaroxidans* DSM 10017 | HNH endonuclease | Sfum_0985 | 107 | ND |
|  |  | | *Syntrophobacter fumaroxidans* DSM 10017 | Endonuclease/exonuclease/phosphatase | Sfum_0613 | 291 | ND |
|  |  |  | *Campylobacter jejuni* RM1221 | DNA/RNA non-specific endonuclease | CJE1441 | 217 | ND |
|  |  | | *Helicobacter pylori* v225d | Endonuclease | HPV225_1419 | 282 | ND |
|  |  | | *Wolinella succinogenes* | Ribonuclease | rnhB | 183 | ND |
|  |  |  | *Acinetobacter baumannii* | Putative extracellular nuclease | A1S_1198 | 298 | ND |
|  |  | | *Aeromonas hydrophila* | Extracellular deoxyribonuclease | dns | 230 | + |
|  |  | | *Aeromonas hydrophila* | Extracellular deoxyribonuclease | N/A | 227 | + |
|  |  | | *Aeromonas salmonicida* (strain A449) | Extracellular nuclease | nucH ASA_2206 | 1072 | - |
|  |  | | *Alteromonas macleodii* DSM17117 | Ribonuclease H | rnhA | 156 | - |
|  |  | | *Alteromonas macleodii* DSM17117 | Ribonuclease | MADE_1011800 | 1346 | ND |
|  |  | | *Azotobacter vinelandii*  ATCC BAA-1303 | Staphylococcus nuclease (SNase-like) | Avin_08950 | 208 | ND |
|  |  | | *Azotobacter vinelandii*  ATCC BAA-1303 | HNH endonuclease domain-containing protein | Avin_17990 | 116 | ND |
|  |  | | *Buchnera aphidicola* | endonuclease-1 | endA | 245 | ND |
|  |  | | *Colwellia psychrerythraea* 34H/ATCC BAA 681 | Extracellular ribonuclease/nuclease fusion protein | CPS_2634 | 1310 | ND |
|  |  | | *E.coli* (K12) | Exodeoxyribonuclease 1 | sbcB | 475 | ND |
|  |  | | *Erwinia tasmaniensis* | Nuclease nucM | nucM | 237 | ND |
|  |  | | *Idiomarina loihiensis* | Extracellular nuclease | IL1523 | 589 | ND |
|  |  | | *Photobacterium profundum* | Extracellular nuclease | PBPRA0287 | 781 | ND |
|  |  | | *Pseudoalteromonas atlantica* | S1/P1 nulcease | - | 256 | - |
|  |  | | *Pseudomonas aeruginosa* | Predicted extracellular nuclease | PA14_13340 | 780 | ND |
|  |  | | *Pseudomonas fluorescens biotypeA* | Extracellular endonuclease | endX | 229 | + |
|  |  | | *Salmonella choleraesuis* | Putative DNA/RNA non-specific nuclease | nucA | 284 | ND |
|  |  | | *Serratia marcescens* | Nuclease | nucA | 266 | + |
|  |  | | *Shewanella oneidensis* | Extracellular nuclease, putative | SO_1844 | 948 | - |
|  |  | | *Shigella flexneri* | Micrococcal nuclease | nuc | 174 | ND |
|  |  | | *Teredinibacter turnerae*  ATCC 39867 | Extracellular nuclease | TERTU_0904 | 796 | ND |
|  |  | | *Vibrio cholerae* | Extracellular nuclease | dns VC-0470 | 231 | + |
|  |  | | *Vibrio parahaemolyticus* | Extracellular nuclease related protein | VP2799 | 984 | ND |
|  |  | | *Xanthomonas campestris* | Extracellular nuclease | XCV0282 | 572 | ND |
|  |  | | *Xanthomonas campestris* B100 | Endonuclease S1 | nucS | 270 | ND |
|  |  | | *Yersinia mollaretti* ATCC 43969 | Nuclease nucM | N/A | 235 | ND |
|  | Aquificae | | *Aquifex aeolicus* | Probable endonuclease 4 | nfo | 282 | - |
|  |  | | *Hydrogenobaculum sp* | HNH endonuclease | HY04AAS1_0070 | 174 | ND |
|  |  | | *Persephonella marina*  DSM 14350 | HNH endonuclease | PERMA_1611 | 195 | ND |
|  | Bacteroidetes | | *Bacteroides fragilis* | Probable endonuclease 4 | nfo | 278 | - |
|  |  | | *Cytophaga hutchinsonii* ATCC 33406 | Nuclease | CHU_1190 | 166 | ND |
|  |  | | *Cytophaga hutchinsonii* ATCC 33406 | Endonuclease/exonuclease/phosphatase family protein | CHU_3419 | 339 | ND |
|  |  | | *Flavobacterium johnsoniae* | Endonuclease/exonuclease/phosphatase | Fjoh_1565 | 279 | ND |
|  |  | | *Flavobacterium psychrophilum* | Probable extracellular ribonuclease | FP0696 | 365 | ND |
|  |  | | *Leeuwenhoekiella blandensis* | Putative S1/P1 Nuclease | MED217_13971 | 263 | ND |
|  |  | | *Prevotella bryantii* | NucB | nucB | 300 | ND |
|  |  | | *Prevotella buccalis* ATCC 35310 | Nuclease. EndA/nucM family | HMPREF0650_1380 | 516 | ND |
|  |  | | *Prevotella melaninogenica* ATCC 25845 | Nuclease, EndA/NucM family | HMPREF0659_A5053 | 500 | ND |
|  | Chlamydiae | | *Chlamydia pneumonia* | Probable endonuclease 4 | nfo | 293 | - |
|  | Chloroflexi | | *Dehalococcoides mccartyi* (strain CBDB1) | Endonuclease V | nfi | 223 | - |
|  |  | | *Herpetosiphon aurantiacus* | DNA/RNA non-specific endonucleas | Haur_1138 | 356 | ND |
|  | Cyanobacteria | | *Nostac sp. Strain* PCC7120 | Nuclease | nucA | 274 | - |
|  |  | | *Synechocystis sp.* (strain PCC 6803) | Extracellular nuclease | nucH | 1879 | - |
|  | Deferribacteres | | *Deferribacter desulfuricans* DSM 14783 | Thermonuclease | DEFDS_P103 | 179 | ND |
|  |  | | *Deferribacter desulfuricans* DSM 14783 | Ribonuclease HII | rnhB | 187 | - |
|  | Deinococcus-Thermus | | *Deinococcus radiodurans* | Endonuclease III | DR_0289 | 225 | - |
|  |  | | *Meiothermus ruber* | Probable endonuclease 4 | nfo` | 266 | - |
|  | Firmicutes | | *B.amyloliquefaciens* | Extracellular ribonuclease | bsn | 289 | + |
|  |  | | *B.amyloliquefaciens* DSM7 | NucB protein | nucB | 144 | ND |
|  |  | | *B.amyloliquefaciens* FZB42 | NucA | nucA | 145 | - |
|  |  | | *Bacillus amyloliquefaciens* FZB42 | NucB | nucB | 144 | + |
|  |  | | *Bacillus atrophaeus* 1942 | Endonuclease | nd | 145 | - |
|  |  | | *Bacillus atrophaeus* 1942 | Nuclease | N/A | 144 | ND |
|  |  | | *Bacillus cereus* AH603 | DNA entry nuclease | - | 144 | - |
|  |  | | *Bacillus cereus anthracis sp.* | Hypothetical nuclease comI family | - | 142 | ND |
|  |  | | *Bacillus cereus* str Q1 | Competence specific | ComI | 148 | ND |
|  |  | | *Bacillus licheniformis* ATCC14580 | Nuclease NucA | nucA | 148 | - |
|  |  | | *Bacillus licheniformis* ATCC14580 | NucB | nucB | 142 | + |
|  |  | | *Bacillus licheniformis* ATCC14580 | Extracellular ribonuclease | yurl/ bsn BLi03441 | 270 | ND |
|  |  | | *Bacillus megatarium* | Competence-specific nuclease | nucA | 146 | - |
|  |  | | *Bacillus megatarium* DSM 319 | Sporulation-specific extracellular nuclease | nucB | 145 | + |
|  |  | | *Bacillus pumilus* ATCC7061 | DNA entry nuclease | N/A | 144 | - |
|  |  | | *Bacillus pumilus* SAFR-032 | Possible sporulation specific | nucB | 142 | + |
|  |  | | *Bacillus subtilis* 168 | DNA entry nuclease | nucA | 147 | - |
|  |  | | *Bacillus subtilis* 168 | Sporulation specific extracellular nuclease | nucB | 136 | + |
|  |  | | *Bacillus subtilis natto* BEST195 | Nuclease | nucA | 149 | - |
|  |  | | *Bacillus subtilis* spiz W23 | Endonuclease | nucA | 145 | - |
|  |  | | *Bacillus subtilis spiz* W23 | Nuclease | nucB | 136 | ND |
|  |  | | *Bacillus thuringensis konkukian sp.* | possible competence specific nuclease | comI | 144 | - |
|  |  | | *Bacillus subtilis* | Extracellular ribonuclease | bsn yurI BSU32540 | 288 | + |
|  |  | | *Brevibacillus brevis* NBRC 10059 | Probable nuclease | BBR47_19480 | 142 | ND |
|  |  | | *Desulfotomaculum acetoxidans* | Endonuclease III | Dtox_0804 | 219 | - |
|  |  | | *Enterococcus faecalis* | Putative uncharacterized protein | N/A | 192 | - |
|  |  | | *Eubacterium eligens* | Endonuclease | EUBELI_20121 | 316 | ND |
|  |  | | *Finegoldia magna*  ATCC 53516 | Endo/exonuclease | N/A | 91 | - |
|  |  | | *Geobacillus sp.*Y412MC10 | Nuclease | GYMC10_5293 | 146 | - |
|  |  | | *Lactobacillus johnsonii* | Extracellular/ cell surface nuclease | endA | 282 | + |
|  |  | | *Paenibacillus polymyxa* SC2 | DNA entry nuclease | N/A | 245 | ND |
|  |  | | *Staphylococcus aureus* | Staphylococcal/micrococcal/thermococcal nuclease | nuc | 231 | Nuc A +,  Nuc B - |
|  |  | | *Staphylococcus aureus* MW2 | Thermonuclease | nuc | 228 | + |
|  |  | | *Staphylococcus hyicus* | Thermonuclease | nucH | 169 | + |
|  |  | | *Staphylococcus intermedius* | Thermonuclease | nucI | 168 | + |
|  |  | | *Streptococcus equi subsp. zooepidemicus* | Extracellular nuclease | Sez_0781 | 926 | + |
|  |  | | *Streptococcus sanguinis* | Extracellular nuclease, putative | SSA_1750 | 749 | ND |
|  |  | | *Thermincol potens* | Endonuclease III | TherJR_2368 | 208 | - |
|  | Fusobacteria | | *Fusobacterium ulcerans* ATCC 49185 | Nuclease | SNc | 156 | ND |
|  |  | | *Fusobacterium nucleatum* ATCC 23726 | Family 2 AP endonuclease | HMPREF0397_0109 | 316 | ND |
|  |  | | *Streptobacillus moniliformis* ATCC 14647 | Endonuclease/exonuclease/phosphatase family | Smon_0995 | 277 | ND |
|  | Nitrospirae | | *Candidatus Nitrospira defluvii* | Putative HNH endonuclease | NIDE2766 | 181 | ND |
|  |  | | *Candidatus Nitrospira defluvii* | Putative Endonuclease | NIDE3656 | 82 | ND |
|  | Planctomycetes | | *Rhodopirellula baltica* | Extracellular nuclease | RB4375 | 3056 | - |
|  |  | | *Candidatus Kuenenia stuttgartiensis* | Highly similar to nuclease | nucI | 175 | ND |
|  |  | | *Cloacamonas acidaminovorans* | Putative micrococcal nuclease | CLOAM1654 | 251 | ND |
|  |  | | *Pirellula staleyi* ATCC 27377 | Nuclease (SNase domain protein) | Psta_2998 | 178 | ND |
|  |  | | *Pirellula staleyi* ATCC 27377 | Endonuclease/exonuclease/phosphatase | Psta_4178 | 290 | ND |
|  | Spirochaetae | | *Borrelia afzelii (strain Pko)* | Endonuclease | nucA | 293 | ND |
|  |  | | *Leptospira biflexa serovar Patoc* | HNH nuclease | LBF_1075 | 97 | ND |
|  |  | | *Leptospira interrogans* | Nuclease-like protein | LIC_10505 | 325 | ND |
|  |  | | *Treponema pallidum* | Endonuclease/exonuclease/phosphatase family | TPChic_0544 | 622 | ND |
|  |  | | *Treponema vincentii* | HNH endonuclease domain protein | TREVI0001_0568 | 172 | ND |
|  | Thermotogae | | *Thermotoga maritime* | Endonuclease V | nfi | 225 | - |
|  | Verrucomicrobia | | *Methylacidiphilum infernorum* | NurA Nuclease | Minf_0851 | 384 | ND |
|  |  | | *Methylacidiphilum infernorum* | Predicted endonuclease | Minf_1271 | 78 | ND |
|  |  | | *Methylacidiphilum infernorum* | HNH nuclease | Minf_0150 | 221 | ND |
|  | Tenericutes | | *Mycoplasma agalactiae* | Nuclease | MAG0790 | 164 | ND |
|  |  | | *Mycoplasma mobile* | Staphylococcal nuclease | nuc | 200 | ND |
|  |  | | *Ureaplasma urealyticum serovar* 10 ATCC 33699 | Endonuclease/exonuclease/phosphatase family protein | UUR10_0061 | 1134 | ND |
|  | Fibrobacteres | | *Fibrobacter succinogenes* ATCC 19169 | Deoxyribonuclease I | endA | 247 | ND |
|  | Gemmatimonadetes | | *Gemmatimonas aurantiaca* DSM 14586 | Endonuclease | GAU_2200 | 285 | ND |
|  | Nitrospirae | | *Thermodesulfovibrio yellowstonii* ATCC 51303 | Thermonuclease | THEYE_A0153 | 183 | ND |
|  | Synergistetes | | *Aminobacterium colombiense* DSM 12261 | Endonuclease/exonuclease/phosphatase | Amico_1146 | 359 | ND |
|  |  | | *Aminobacterium colombiense* DSM 12261 | Nuclease (SNase domain protein) | Amico_1854 | 264 | ND |
|  |  | | *Anaerobaculum hydrogeniformans* ATCC BAA-1850 | Thermonuclease | nucH | 282 | ND |
|  |  | | *Anaerobaculum hydrogeniformans* ATCC BAA-1850 | Endonuclease/exonuclease/phosphatase family protein | HMPREF1705_00741 | 240 | ND |
|  |  | | *Anaerobaculum hydrogeniformans* ATCC BAA-1850 | AP endonuclease, family 2 superfamily | HMPREF1705_02136 | 268 | ND |
|  |  | | *Dethiosulfovibrio peptidovorans* DSM 11002 | Nuclease (SNase domain protein) | Dpep_1102 | 258 | ND |
|  | Dictyoglomi | | *Dictyoglomus thermophilum* ATCC 35947 | HNH endonuclease domain protein | DICTH_0738 | 166 | ND |
| **Archaea** | Crenarchaeota | | *Sulfolobus solfataricus* | Flap endonuclease 1 | fen | 349 | ND |
|  |  | | *Ignicoccus hospitalis* DSM 18386 | Endonuclease (RecB family)-like protein | Igni_0481 | 195 | ND |
|  |  | | *Ignicoccus hospitalis* DSM 18386 | Nuclease (SNase domain protein) | Igni_1343 | 227 | ND |
|  | Euryarchaeota | | *Methanobrevibacter ruminantium* ATCC 35063 | Hef nuclease | mru_2089 | 842 | ND |
|  |  | | *Methanobrevibacter smithii* ATCC 35061 | Nuclease, Staphylococcus nuclease-like family | Msm_1495 | 161 | ND |
|  |  | | *Thermococcus onnurineus* | Staphylococcus nuclease | TON_1652 | 152 | ND |
|  |  | | *Thermoproteus neutrophilus* DSM 2338 | Secreted endonuclease-like protein | Tneu_1017 | 215 | + |
|  |  | | *Methanosarcina acetivorans* | Hnh endonuclease | MA_3494 | 279 | ND |
|  |  | | *Halobacterium marismortui* | putative nuclease | rrnAC2569 | 94 | ND |
|  |  | | *Methanococcus maripaludis* | Staphylococcus nuclease (SNase-like):Thermonuclease | MMP1201 | 185 | ND |
|  |  | | *Thermoplasma sp.* P61 | Flap endonuclease-1 | fen-1 | 328 | ND |
|  | Korarchaeota | | *Korarchaeum cryptofilum* | predicted nuclease | Kcr_0633 | 183 | ND |
|  | Nanoarchaeota | | *Nanoarchaeum equitans* Kin4-M | Flap structure-specific endonuclease | fen | 339 | ND |
|  | Thaumarchaeota | | *Nitrosopumilus maritimus* SCM1 | Ribonuclease HII | rnhB | 205 | - |
|  |  | | *Cenarchaeum symbiosum* | Micrococcal nuclease-like protein | CENSYa_0688 | 637 | ND |
|  |  | | *Cenarchaeum symbiosum* | HNH nuclease | CENSYa_1878 | 338 | ND |
| **Fungi** | Ascomycota | | *Aspergillus niger* | nuclease | N/A | 334 | ND |
|  |  | | *Aspergillus oryzae* | Nuclease S1 | nucS | 287 | ND |
|  |  | | *Aspergillus fumigatus* | Nuclease protein domain | BA78_0190 | 296 | ND |
|  |  | | *Penicillum citrinum* | Nuclease P1/ Dnase P1/Endonuclease P1 | N/A | 270 | + |
|  |  | | *Penicillium sp.* | Nuclease PA3 | N/A | 270 | + |
|  |  | | *Candida albicans* | Endo-exonuclease NUCR | CAWG_01238 | 551 | ND |
|  |  | | *Candida dubliniensis* | nuclease, putative | N/A | 235 | ND |
|  |  | | *Candida tropicalis* | mitochondrial nuclease | CTRG_05252 | 375 | ND |
|  |  | | *Schizosaccharomyces pombe* | AP endonuclease 2 | apn2 | 523 | - |
|  |  | | *Syncephalastrum racemosum* | Nuclease/ Sr-nuclease | N/A | 320 | + |
|  | Zygomycota | | *Cunninghamella echinulata var. echinulata* | Nuclease C1 | NUC1CE | 252 | + |
|  |  | | *Rhizopus niveus* | Ribonuclease Rh | N/A | 238 | ND |
| **Eukaryota** | Chlorophyta | | *Ostreococcus tauri* | probable extracellular nuclease | N/A | 1490 | + |
|  |  | | *Naegleria gruberi* (Amoeba) | probable extracellular nuclease | NAEGRDRAFT_78311 | 1888 | - |
|  | Percolozoa | | *Plasmodium yoelii yoelii* | AP endonuclease 1 | PY05725 | 460 | - |
|  | Apicomplexa | | *Enterobacteri phage* T15 | Exodeoxyribonuclease | D15 | 291 | ND |
| **Virus** |  | | *Human herpesvirus 4* | Shutoff alkaline exonuclease | BGLF5 | 470 | ND |
|  |  | |  |  |  |  |  |
